# Effect of hysterectomy on re-operation for stress urinary incontinence: 10 year follow-up

**DOI:** 10.1007/s00404-022-06737-4

**Published:** 2022-08-31

**Authors:** Sari Tulokas, Maarit Mentula, Päivi Härkki, Tea Brummer, Tea Kuittinen, Tomi Mikkola, Päivi Rahkola-Soisalo

**Affiliations:** 1 Department of Obstetrics and Gynaecology, University of Helsinki and Helsinki University Hospital, Helsinki, Finland; 2 Central Hospital Østfold, Fredrikstad, Norway

**Keywords:** Stress urinary incontinence, Hysterectomy, Mid-urethral sling

## Abstract

**Purpose:**

Hysterectomy and mid-urethral sling (MUS) are common operations, but little is known about how hysterectomy after MUS affects the risk for stress urinary incontinence (SUI) relapse.

**Methods:**

We included 49 women with a MUS before hysterectomy and 41 women with a MUS concomitant with hysterectomy. The controls, matched by age (± 2 years), MUS type (retropubic vs transobturator) and operation year (± 2 years), included 201 women who underwent the MUS operation without a subsequent hysterectomy. We used health care registers for follow-up of 12.4 years in median (IQR 10.9–14.7) after the MUS operation to compare the number of SUI re-operations and hospital re-visits for urinary incontinence.

**Results:**

The re-operation rates for SUI did not differ between the women with MUS before hysterectomy (*n* = 2, 4.1%), women with MUS concomitant with hysterectomy (*n* = 2, 4.9%) and their controls (*n* = 4, 4.9%, *p* = 0.8 and *n* = 6, 5.0%, *p* = 1.0, respectively). There were significantly fewer urinary incontinence re-visits among women who had a MUS concomitant with the hysterectomy compared to their matched controls (*n* = 2 and 31, 5 and 31%, *p* < 0.01) and to the women with a MUS prior to hysterectomy (*n* = 2 and 10, 5 and 20%, respectively, *p* = 0.03).

**Conclusion:**

Hysterectomy after or concomitant with MUS does not seem to increase the risk for SUI re-operation or hospital re-visits for urinary incontinence. These results can be used to counsel women considering hysterectomy after MUS operation or concomitant with MUS operation.

## What does this study add to the clinical work

**Table:** 

Performing hysterectomy after or concomitant with a mid-urethral sling operation does not seem to increase the rate of re-operation for stress urinary incontinence.	

## Introduction

Hysterectomy and mid-urethral sling (MUS) operations are both common: 30% of women in the United States undergo a hysterectomy by the age of 60 [1, 2] and 6–10% undergo a MUS operation during their lifetime [3, 4]. While hysterectomy improves pre-existing urinary incontinence (UI) symptoms in half of women [5, 6], it increases the overall risk for stress urinary incontinence (SUI) [7–10], in particular for those who have a pelvic organ prolapse (POP) or undergo a vaginal hysterectomy [11]. In contrast, the effect of hysterectomy on SUI relapse risk after a previous MUS operation remains unknown.

In Finnish clinical practice, women with SUI and an indication for hysterectomy would likely be hysterectomized first, possibly with a concomitant MUS. However, the need for hysterectomy may appear later in life after a MUS operation. When MUS is combined with a POP operation for women with prior SUI, postoperative SUI and the need for a later SUI operation are reduced [12–14], but studies assessing hysterectomy after MUS are lacking.

In this retrospective case–control study, we compared the long-term risk for SUI re-operation after MUS in women with a subsequent or concomitant hysterectomy to women without a hysterectomy. As primary outcome, we used re-operations for SUI, and as secondary outcome, hospital re-visits for UI.

## Materials and methods

We selected the cases from a previous national FINHYST 2006 cohort study of 5279 women who underwent a hysterectomy for a benign indication in Finland 2006. All women provided a written informed consent and gave permission for further analyses. Baseline data were collected in 2006 by gynecological surgeons as previously described in detail [15]. Women were included as cases if they had a concomitant MUS operation recorded in the FINHYST data or a prior or concomitant MUS operation (NCSP codes LEG10 for retropubic MUS, LEG12 and LEG13 for transobturator MUS) according to the national Register for Health Care (Care Register). This register maintained by the Finnish Institute for Health and Welfare contains diagnoses and operation codes for all in- and outpatient visits in every private and public hospital in Finland. The validity of the Care Register with respect to different medical conditions has been confirmed in previous studies [16, 17]. To identify women with POP before hysterectomy, we identified case women who had a hospital visit for POP (diagnoses code ICD-10 N81*) or a POP operation (NCSP code LEF*) reported in the Care Register.

Our control group consisted of women who had a MUS operation in the Helsinki University Hospital region between 1998 and 2006. The controls were matched by age (± 2 years), operation type (retropubic MUS vs transobturator MUS), and operation year (± 2 years). We identified control women with hysterectomies from the hospital records. Women with a hysterectomy before the index MUS or unavailable information on hysterectomy were excluded.

Our main outcome was SUI re-operation, which we defined as a visit with a SUI operation code (NCSP codes LEG*, KDG*, KDV20 and KDV22) in the Care Register from 60 days after the index MUS until the end of 2016. Our secondary outcome was UI re-visits, which we defined as a visit with a UI diagnosis code (ICD-10 N39.3 for SUI, and N39.4 for other UI) in the Care Register from 60 days after the index MUS until the end of 2016. Only the first operation for SUI or visit for UI was reported for each woman. The indication of index MUS and SUI re-operation was defined as SUI for ICD-10 diagnoses code N39.3 and as other UI for any other diagnoses code. Other UI as MUS indication includes mostly women with stress-dominant mixed urinary incontinence, because MUS is used mainly to treat SUI and stress-dominant mixed urinary incontinence in Finland. We stopped the follow-up at the end of 2016 or at a hysterectomy in the control group.

To compare groups, we used the Student’s t test for continuous variables, the chi-squared or Kruskal–Wallis test, when appropriable, for categorical variables. We used an odds ratio (OR) to assess the association with re-operations and re-visits. In a sub-analysis, we divided the data into subgroups according to the MUS timing: prior to or concomitant with the hysterectomy. IBM SPSS Statistics 27 was used for statistical analysis. A significance level of *p* < 0.05 was used. We used Kaplan–Meier to estimate cumulative survival without a re-visit for UI.

The study protocol was approved by the Ethical Committee of the Helsinki and Uusimaa Hospital District (Dnro 457/E8/04 and 343/13/03/03/2015) and was registered in the Clinical Trials (NCT00744172). The Finnish Institute for Health and Welfare of Finland authorized the use of the data from the Care Register (THL/986/5.05.00/2018).

## Results

**Fig. 1:**
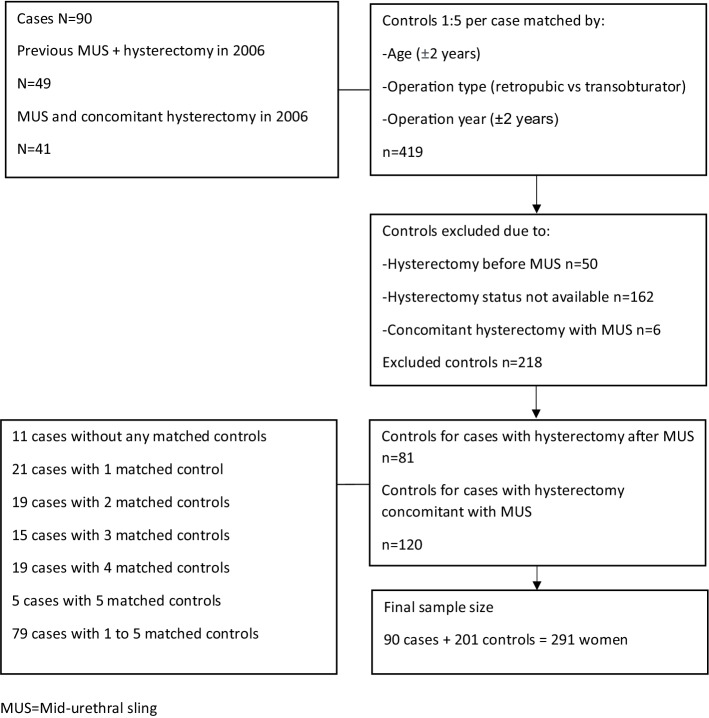
Flowchart

We identified 49 women with a MUS prior to a hysterectomy and 41 women with a MUS concomitant with a hysterectomy. We identified 419 women as matched controls, but fifty of them were excluded for having a hysterectomy before MUS, 162 for unknown hysterectomy history, and six women for hysterectomy concomitant with MUS, leaving a total of 201 controls (Fig. 1).

When performed concomitant with hysterectomy, a transobturator MUS was most often selected as the sling type (*n* = 29, 71%). In the subgroup of MUS before hysterectomy, there were more multiparas in the case group than in the control group (*n* = 43 and 52, 48 and 26%, *p* = 0.05, respectively). The most common main indication for hysterectomy was POP (37%), and a concomitant POP operation was performed in 33% (*n* = 30) of the cases (Tables 1, 2).

**Table 1:** Patient demographics

	All patients (291)			MUS before hysterectomy (130)			MUS concomitant with hysterectomy (161)		
	Cases (90)	Controls (201)	*p*	Cases (49)	Controls (81)	*p*	Cases (41)	Controls (120)	p
Age at MUS, mean (+ -SD)	50.9 (9.8)	48.9 (8.5)	0.07	50.3 (9.6)	45.1 (7.2)	0.09	51.7 (10.1)	50.1 (9.1)	0.39
Age at hysterectomy, mean (+ -SD)	52.9 (9.9)	–	–	53.9 (9.7)	–		51.7 (10.1)	–	
MUS type, n (%)			0.06			0.41			0.83
Retropubic MUS	60 (66.7)	110 (54.7)		48 (98.0)	77 (95.1)		12 (29.3)	33 (27.5)	
Transobturator MUS	30 (33.3)	91 (45.3)		1 (2.0)	4 (4.9)		29 (70.7)	87 (72.5)	
MUS year			0.9			0.001			1.0
1998–2000	7 (7.8)	25 (12.4)		7 (14.3)	25 (30.9)		0 (0)	0 (0)	
2001–2003	27 (30.0)	49 (24.4)		27 (55.1)	49 (60.5)		0 (0)	0 (0)	
2004–2006	56 (62.2)	127 (63.2)		15 (30.6)	7 (8.6)		41 (100.0)	120 (100.0)	
MUS indication			0.01			0.15			0.21
Stress urinary incontinence	67 (74.4)	118 (58.7)		45 (91.8)	67 (82.7)		22 (53.7)	51 (42.5)	
Other	23 (25.6)	83 (41.3)		4 (8.2)	14 (17.3)		19 (46.3)	69 (57.5)	
Previous deliveries			0.07			0.05			0.43
Nullipara or unknown	9 (10.0)	41 (20.4)		4 (8.2)	13 (16.0)		5 (12.2)	28 (23.3)	
1 to 2	38 (42.2)	108 (53.7)		22 (44.9)	51 (63.0)		16 (39.0)	57 (47.5)	
3 or more	43 (47.8)	52 (25.9)		23 (46.9)	17 (21.0)		20 (48.8)	35 (29.2)	

*MUS*  mid-urethral sling

**Table 2:** Hysterectomy indication, method, and concomitant operations

	All hysterectomies (90)	MUS before hysterectomy (49)	MUS concomitant with hysterectomy (41)	*p*
Hysterectomy timing				
Time from MUS to hysterectomy, median in years (IQR)	–	3.7 (2.1–5.0)	–	–
Hysterectomy main indication				0.35
Fibroids	21 (23.3)	14 (28.6)	7 (17.1)	
Menorrhagia	18 (20.0)	6 (12.2)	12 (29.3)	
Dysmenorrhea	4 (4.4)	2 (4.1)	2 (4.9)	
Endometriosis	1 (1.1)	0 (0)	1 (2.4)	
POP	33 (36.7)	16 (32.7)	17 (41.5)	
Adnex tumor	6 (6.7)	4 (8.2)	2 (4.9)	
Other	7 (7.8)	7 (14.3)	0 (0)	
Hysterectomy method				0.11
Abdominal	15 (16.7)	13 (26.5)	2 (4.9)	
Vaginal	57 (63.3)	26 (53.1)	31 (75.6)	
Laparoscopic	18 (20.0)	10 (20.4)	8 (19.5)	
Concomitant POP operations with hysterectomy				
Any	30 (33.3)	16 (32.7)	14 (34.1)	0.88
Anterior colporrhaphy	23 (25.6)	11 (22.4)	12 (29.3)	0.47
Posterior colporrhaphy	18 (20.0)	9 (18.4)	9 (22.0)	0.68
Enterocele	3 (3.3)	2 (4.1)	1 (2.4)	0.67
Other	1 (1.1)	1 (2.0)	0 (0)	0.36
Preceding POP^a^	46 (51.1)	22 (44.9)	24 (58.5)	0.2
Concomitant sacrospinous fixation with hysterectomy,* n* (%)	1 (1.1)	0 (0)	1 (2.4)	0.28

*MUS*  mid-urethral sling operation, *POP* pelvic organ prolapse

^a^Main indication POP, concomitant POP operation, or preceding POP diagnoses/operation

There was no difference in the SUI re-operation rates between cases and controls when analyzing all women (4.4 and 5.0%, *p* = 0.9) or when analyzing sub-groups of women with MUS prior to hysterectomy (4.1 and 4.9%, *p* = 0.8) or MUS concomitant with hysterectomy (4.9 and 5.0%, *p* = 1.0). Most of the re-operations were for SUI in both cases and controls (*n* = 3 and 7, 75 and 70%, Table 3). There were no cases that had a re-operation for MUS before the hysterectomy, but five cases had a hospital visit for UI between the index MUS and before the subsequent hysterectomy, two for SUI and three for other UI. The re-operations for SUI consisted of five retropubic MUS operations, two transobturator MUS operations, one other vaginal operation for incontinence and six other operations on urethra or bladder neck for incontinence.

**Table 3:** Re-operations for stress urinary incontinence and re-visits for urinary incontinence

	All women (291)			MUS before hysterectomy (130)			MUS concomitant with hysterectomy (161)		
	Cases (90)	Controls (201)	OR (95% CI)	Cases (49)	Controls (81)	OR (95% CI)	Cases (41)	Controls (120)	OR (95% CI)
Follow-up time, median in years (IQR)	11.6 (10.6–14.2)	12.0 (10.4–14.1)		14.0 (12.9–15.5)	14.7 (12.3–16.3)		10.6 (10.3–10.9)	11.5 (10.2–12.2)	
Hysterectomy before the end of follow-up	–	30 (14.9)		–	19 (23.5)		–	11 (9.2)	
Stress urinary incontinence re-operation	4 (4.4)	10 (5.0)	0.9 (0.3–2.9)	2 (4.1)	4 (4.9)	0.8 (0.1–4.6)	2 (4.9)	6 (5.0)	1.0 (0.2–5.0)
Retropubic MUS	1 (25.0)	4 (40.0)		1 (50.0)	0 (0.0)		1 (50.0)	4 (66.7)	
Transobturator MUS	2 (50.0)	0 (0.0)		1 (50.0)	0 (0.0)		1 (50.0)	0 (0.0)	
Urethral injections	0 (0.0)	0 (0.0)		0 (0.0)	0 (0.0)		0 (0.0)	0 (0.0)	
Other SUI re-operations	1 (25.0)	6 (60.0)		0 (0.0)	4 (100.0)		0 (0.0)	2 (33.3)	
Time between index MUS and SUI re-operation, median in years (IQR)	4.3 (1.3–7.0)	1.7 (0.5–14.5)		5.7 (3.6-NA)	14.5 (5.4–15.1)		2.7 (0.6-NA)	0.8 (0.4–1.6)	
SUI re-operation indication, *n* (%)									
Stress urinary incontinence	3 (75.0)	7 (70.0)		2 (100.0)	2 (50.0)		1 (50.0)	5 (83.3)	
Other urinary incontinence	1 (25.0)	3 (30.0)		0 (0.0)	2 (50.0)		1 (50.0)	1 (16.7)	
Urinary incontinence re-visit, n (%)	12 (13.3)	57 (28.4)	0.4 (0.2–0.8)	10 (20.4)	26 (32.1)	0.5 (0.2–1.3)	2 (4.9)	31 (25.8)	0.1 (0.03–0.6)
Re-visits before hysterectomy	5 (41.7)	–		5 (50.0)	–		–	–	
Stress urinary incontinence	6 (50.0)	36 (63.2)		5 (50.0)	10 (38.5)		1 (50.0)	26 (83.9)	
Other urinary incontinence	6 (50.0)	21 (36.8)		5 (50.0)	16 (61.5)		1 (50.0)	5 (16.1)	
Time between index MUS and UI re-visit, median in years (IQR)	5.9 (3.5–7.8)	0.4 (0.2–4.9)		6.8 (4.5–8.2)	2.7 (0.3–10.6)		1.9 (0.2-NA)	0.2 (0.2–0.7)	
Time between hysterectomy to UI re-visit, median in years	2.2 (0.5–4.6)	–		2.5 (0.5–4.8)	–		1.9 (0.2-NA)	–	

*MUS *mid-urethral sling, *IQR* interquartile range, *SUI* stress urinary incontinence, *UI* urinary incontinence, *NA* not available

There were less UI re-visits among women who had MUS concomitant with the hysterectomy compared to controls (*n* = 2 and 31, 5 and 31%, *p* < 0.01) and to the women with a MUS prior to the hysterectomy (*n* = 2 and 10, 5 and 20%, respectively, *p* = 0.03). Both women who had a re-visit for UI after MUS concomitant with the hysterectomy were treated with a new MUS. There were no differences in UI re-visits between women who had a MUS prior to hysterectomy and their controls (*n* = 10 and 26, 20 and 31%, respectively, *p* = 0.1). The UI re-visits among the cases occurred up to 9.3 years after index MUS, after which no re-visits occurred. In the control group, 34 (60%) of the UI re-visits occurred already within the first post-operative year, but the re-visits continued to occur throughout the follow-up time.

In the case group, there was no significant difference in the risk for SUI re-operation or UI re-visit between women with POP as the main indication for hysterectomy (OR 0.6 and 1.9, 95% CI 0.06–5.6 and 0.6–6.4) or a concomitant POP operation with hysterectomy (OR 0.7 and 2.3, 95% CI 0.07–6.7 and 0.7–7.7). There was no difference in the rate of SUI re-operations (*p* = 0.8) or UI re-visits (*p* = 0.5) between the different hysterectomy approaches. Also, the approach for hysterectomy did not affect the risk for SUI re-operation (*p* = 0.8) or UI re-visits (*p* = 0.5).

## Discussion

Based on our results, hysterectomy after MUS does not seem to affect the risk for a SUI re-operation. The overall SUI re-operation rate did not differ between the groups, and it is comparable to the long-term SUI re-operation rate reported previously from Finland [18].

To our knowledge, there are no previous studies focusing on the effect of hysterectomy after MUS. Hysterectomy alone has been associated with an increased risk for SUI operation (1–3) but, in case of pre-operative UI, hysterectomy has been reported to improve the symptoms in 45% of cases (10). Several studies have shown good long-term efficacy rates after MUS operations [19], but they have not reported post-operative hysterectomies. As a secondary result, one study showed a twice higher rate of symptomatic SUI after MUS in women with a subsequent hysterectomy [20]. However, only eight women with a subsequent hysterectomy were included in this study and thus, no decisive conclusions can be drawn.

Cases in our study had fewer UI re-visits than the controls, especially when MUS was performed concomitant with the hysterectomy. Because the failure risk of MUS is higher in women with mixed urinary incontinence compared to SUI [21, 22], a higher proportion of women with SUI could partly explain this difference. However, it is also possible that women with UI benefit from the construction of the pelvic floor support during hysterectomy.

Previous studies have shown MUS concomitant with POP surgery, including hysterectomy, to reduce post-operative SUI [12]. Our findings suggests that MUS is also effective concomitant with hysterectomy for indication other than POP. Even though the lack of preoperative physical evaluation may have prevented us from identifying possible mild POP, we found that only half of the case women had either POP as the main indication for hysterectomy or a concomitant POP operation with the hysterectomy or a previous hospital visit for POP. Furthermore, POP as the main indication for hysterectomy or a concomitant POP operation with hysterectomy did not affect the risk for SUI re-operation or UI re-visit. Unfortunately, we were unable to assess if concomitant MUS and hysterectomy was associated with a higher risk for adverse events, as has been suggested with MUS operations concomitant with POP surgery [14].

As a strength of this study, our sample size is relatively large when considering the methodological difficulties to study the effect of hysterectomy after MUS. Furthermore, the data comes from a high-quality national register with a validated high accuracy rate [16], and we had a long follow up time of over ten years. Thus, we likely recorded most of the relapses, since they usually take place within 5 years after MUS [18, 19]. We also included all benign indications for hysterectomy instead of only POP making the results easier to apply to clinical practice.

As limitations, we acknowledge the retrospective study design that predisposes for confounding factors. The SUI re-operation rate and UI re-visits may also underestimate the symptomatic SUI because not all women seek treatment after relapse. In addition, we were unable to assess the possible adverse events related to the MUS operations.

To conclude, hysterectomy after or concomitant with MUS does not seem to jeopardize the MUS results. These results can be used to counsel women considering hysterectomy after MUS operation or concomitant with MUS operation.

## Data Availability

The data and materials are not available.

## Code availability

IBM SPSS Statistics 27 was used for statistical analysis.
